# Resolution of Cancer-Promoting Inflammation: A New Approach for Anticancer Therapy

**DOI:** 10.3389/fimmu.2017.00071

**Published:** 2017-02-02

**Authors:** Qi Zhang, Bo Zhu, Yongsheng Li

**Affiliations:** ^1^Institute of Cancer, Xinqiao Hospital, Third Military Medical University, Chongqing, China

**Keywords:** inflammation, cancer, lipoxins, resolvins, immunity

## Abstract

Inflammation is a protective response that eliminates harmful stimuli and restores tissue homeostasis, whereas the failure to resolve inflammation leads to the development of malignancies. Immune cells in the tumor inflammatory microenvironment endow cancer cells with their specific hallmarks, including mutations, metabolic reprograming, angiogenesis, invasion, and metastasis. Targeting the inflammatory microenvironment with anti-inflammatory drugs (e.g., aspirin) or by enhancing antitumor immunity (e.g., chimeric antigen receptor T cell therapy) has been extensively investigated and has achieved promising results in many cancers. Recently, a novel approach promoting antitumor immunity *via* a dual anti-inflammatory and pro-resolving strategy was proposed based on the discovery of potent, endogenous, specialized pro-resolving mediators, including lipoxins, resolvins, protectins, and maresins. In this review, we describe the updated principal cellular and molecular mechanisms of inflammation resolution and cancer immunity and discuss the pro-resolution strategy in cancer treatment and prevention.

## Introduction

**Figure 1:**
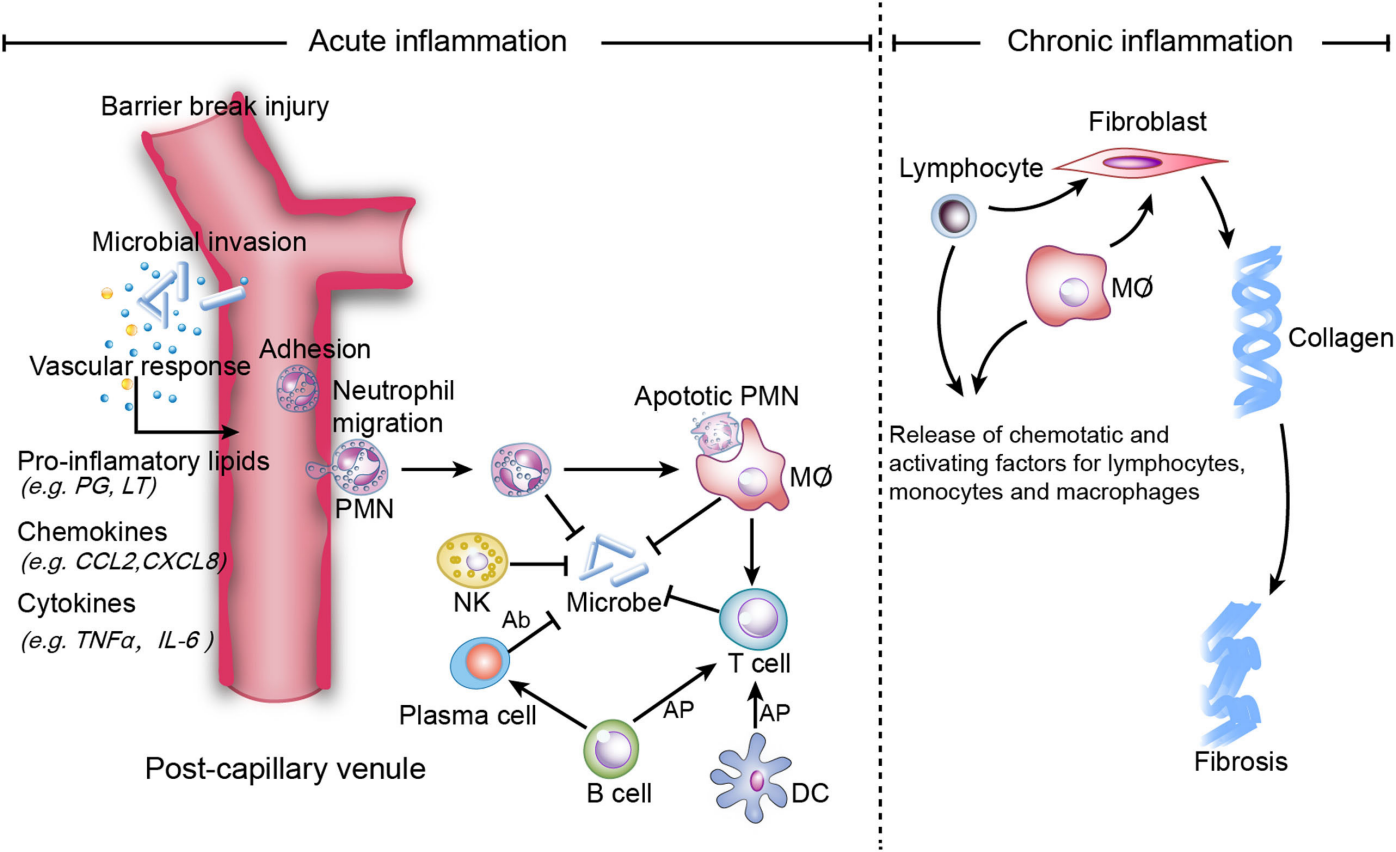
**Mechanisms of acute and chronic inflammation**. Within a few hours of stimulation (injury, trauma, stress, or infection), the release of pro-inflammatory lipids (e.g., prostaglandin (PG), leukotriene (LT), involved in vasodilation), chemokines (e.g., C-C motif chemokine ligand 2 (CCL2), C-X-C motif ligand 8 (CXCL8), involved in chemotaxis and adhesion), and cytokines [e.g., Tumor necrosis factor-α (TNF-α), interleukin (IL)-6] elicits the recruitment of neutrophils. Other immune cells [i.e., natural killer (NK) cells, macrophages, dendritic cells (DCs), B cells, and T cells] also participate in the process. NK cells kill microbes *via* complement-dependent cytotoxicity. Macrophages directly phagocytize organisms and apoptotic neutrophils, while B cells are converted to plasma cells to kill organisms *via* secreted antibodies, which are referred to as antibody-dependent cell-mediated cytotoxicity. Macrophages, B cells and DCs activate T cells *via* antigen cross presentation (AP). Homeostasis will be restored if inflammation is resolved completely, while non-resolution leads to chronic inflammation, which is characterized by persistent tissue infiltration by immune cells (e.g., macrophages, lymphocytes). In the extracellular zone, lymphocytes and macrophages release factors that result in the deposition of extracellular collagen and an excessive inflammatory response.

Inflammation is the protective immune response of a vascular organism that aids in the removal of internal and external harmful stimuli and the maintenance of tissue homeostasis (1). Acute inflammation seeks to repair injured tissues and eliminate unwanted elements. The ideal outcome of acute inflammation is complete and timely resolution with a return to homeostasis, which is actively programed by specialized pro-resolving mediators (SPM), including lipoxins (LXs), resolvins (Rvs), protectins, and maresins (MaRs) (1). SPM potently inhibit neutrophil infiltration and promote macrophage efferocytosis of apoptotic neutrophils in the inflammatory loci (2). However, persistent inflammation leads to chronic inflammation, which is categorized as either delayed-resolving or non-resolving (Figure 1). The symptoms and signs of chronic inflammation are not as serious as those of acute inflammation, but chronic inflammation is typically more risky since it can cause further damage (e.g., fibrosis, necrosis, organ dysfunction, and gene mutation) and an enormous proportion of refractory diseases [e.g., Alzheimer’s disease (AD) (3) and cancer (4)].

As early as the 1860s, Virchow indicated a link between cancer and inflammation by observing inflammatory cells in biopsied tumor tissues (4). Inflammatory stimuli, such as chronic infections, inhaled pollutants, smoking, and obesity (5), may result in DNA damage, somatic mutations, and tumorigenesis (6, 7). In the tumor microenvironment (TME), inflammatory cells are educated to accelerate cancer progression, metastasis, and immune responses against radiotherapy, chemotherapy, and immunotherapy (8). Therefore, targeting the inflammatory microenvironment is a reasonable direction for cancer treatment.

Indeed, anti-inflammatory drugs have exhibited efficacy by improving both prognosis and survival of patients (9, 10) and in cancer prevention (11). Enhancing tumor immunity by blocking inhibitory checkpoints or using chimeric antigen receptor T cell (CAR-T) immunotherapy has also shown promising efficacy in specific cancer types. However, the side effects of these therapies, such as coagulopathy and the “cytokine storm,” have hindered their full application to cancer therapy. Consequently, a better endogenous mechanism for improving the tumor inflammatory microenvironment is urgently needed.

Specialized pro-resolving mediator-driven inflammation resolution is an active process, which results in catabasis and homeostasis. To date, endogenous SPM have been applied in multiple models of cancer and achieved promising outcomes (12–15). In the present review, we highlight the role of inflammation in cancer development (e.g., tumor immunoediting) and suggest the immunomodulatory potential of SPM for cancer treatment in light of a brand-new strategy to remodel the TME by promoting inflammation resolution.

## Cancer-Promoting Inflammation

It has been well established that pathogen-induced inflammation is a high-risk factor for cancer. For instance, persistent *Helicobacter pylori* infection is highly associated with gastric adenocarcinoma and lymphoma (16), human papilloma virus infection increases the risk of cervical cancer (17), hepatitis B and C virus infections increase the incidence of hepatocellular carcinoma (HCC) (18), and infection with Epstein–Barr virus is closely related to nasopharyngeal carcinoma (19). These causative agents lead to persistent infections associated with low levels of chronic inflammation. In addition, some autoimmune diseases also correlate with cancer development. Crohn’s disease and ulcerative colitis, also known as inflammatory bowel diseases, are highly associated with an increased risk of colorectal cancer (CRC) (20). Long-term exposure to irritants or obesity also induces tumor-promoting inflammation (21–23). Senescence-associated inflammation is postulated to be another promoter of most solid malignances (18). Moreover, cancer therapy (e.g., chemotherapy and radiotherapy)-induced inflammation can enhance antigen cross presentation and initiation of the antitumor immune response, whereas these therapies can also initiate inflammation by causing massive necrosis of malignant cells and pericarcinous tissue followed by tumor recurrence and resistance to therapy (18).

**Figure 2:**
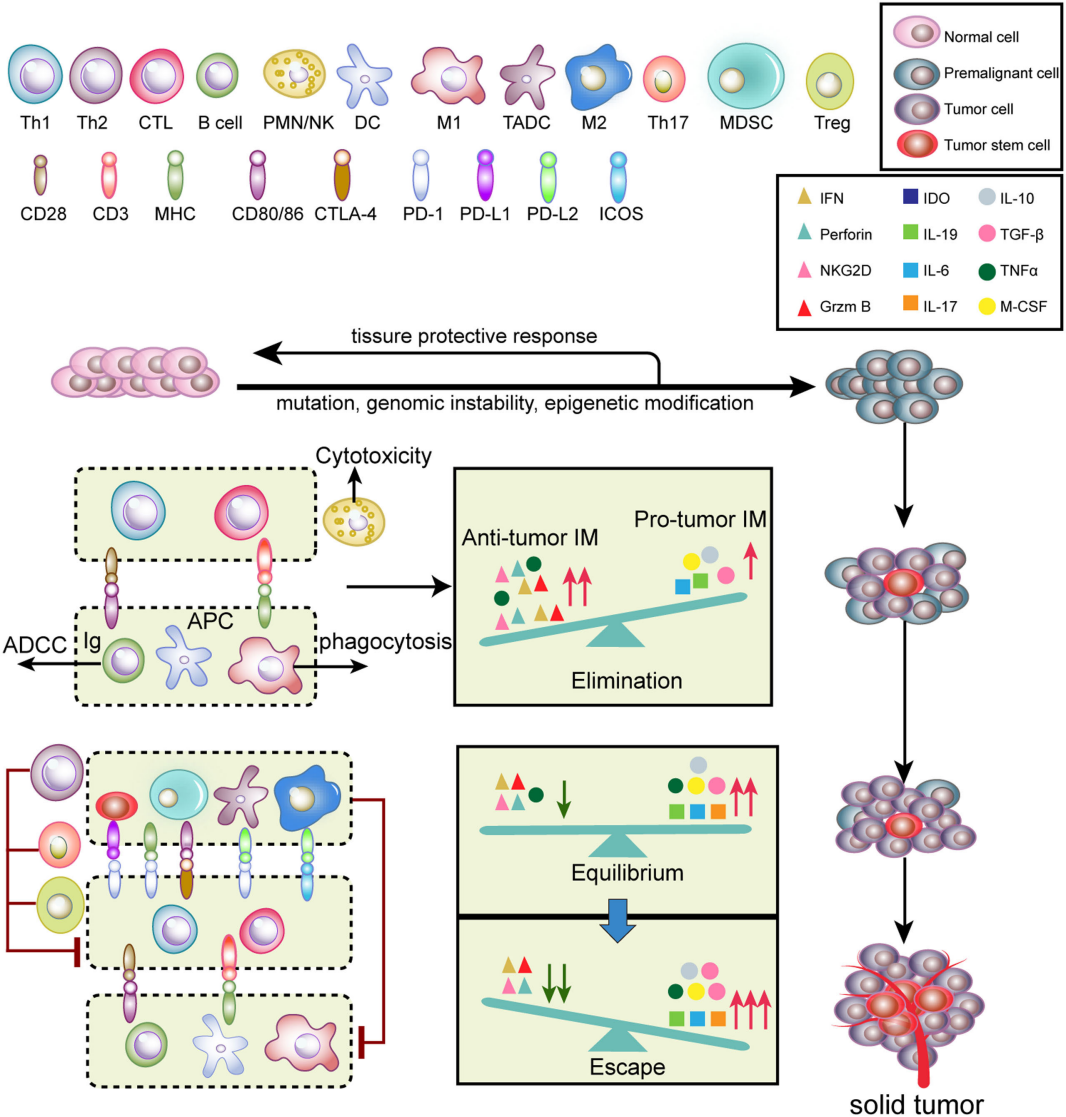
**Tumor immunoediting**. Normal cells are transformed into malignant cells by mutations, genomic instability, and epigenetic modification, during which innate and adaptive immunity regulate the tumor microenvironment. In the elimination phase, both innate and adaptive immunity synergistically detect and eliminate early tumor cells. Next, rare tumor cells that are not eliminated in the elimination phase can enter the equilibrium phase, where their outgrowth and elimination are controlled. Finally, the remaining tumor cell variants with weak immunogenicity escape from immune surveillance to form a clinically apparent neoplasm.

Tumor initiation and progression are finely immunoedited (24). Tumor immunoediting is divided into three phases (Figure 2): elimination, equilibrium, and escape (25). During the elimination phase, tumor cells with potent immunogenicity are removed by the immune system before they become clinically detectable. Activated NK cells and macrophages produce interferon (IFN)-γ and interleukin (IL)-12, which eliminate tumor cells by initiating cytotoxic responses, such as perforin, TNF-α and reactive oxygen species (ROS) (25). Antigen-presenting cells [such as DCs, macrophages, and B cells] take up and cross-present tumor antigens to T cells and activate T cells *via* co-stimulatory molecules (26). Therefore, antitumor inflammatory mediators (IM) predominantly participate in the elimination phase compared to pro-tumor IM. When a balance between pro-tumor and antitumor IM is established, tumors progress into the equilibrium stage. During this phase, variants that survived the elimination phase undergo various mutations but exhibit a weak-immunogenic phenotype (e.g., loss of antigenic tumor peptides and major histocompatibility complex components). Notably, some antitumor cytokines, such as TNF-α, become pro-tumorigenic. This phase may last for several years until new immune-resistant variants emerge, which are more likely to escape immunosurveillance (25). In this scenario, the IM balance is skewed toward pro-tumor IM since immunity fails to limit tumor outgrowth. The immune-resistant variants ultimately result in the formation of a clinically detectable solid tumor (25). In the tumor escape phase, pro-tumor immune cells, including myeloid-derived suppressor cells (MDSCs), tumor-associated dendritic cells (TADCs), tumor-associated macrophages (TAMs), Th17, and regulatory T cells (Tregs), along with cancer cells and cancer stem cells, induce immunosuppression *via* secretion of a variety of immunosuppressive cytokines and molecules. Furthermore, T cells express inhibitory checkpoint receptors, such as programed cell death protein 1 (PD-1) and cytotoxic T-lymphocyte-associated protein 4 (CTLA-4), which are activated by ligands expressed on pro-tumor immune cells (27). Altogether, these immunosuppressive mechanisms synergistically neutralize antitumor immunity and accelerate tumor progression.

The typical underlying mechanisms through which inflammation promotes cancer include (1) mutations: DNA damage/mutation, genomic instability, epigenetic dysregulation, and DNA repair deficiency (28–30). DNA damage in turn promotes inflammation, generating a vicious cycle that synergistically initiates carcinogenesis (28); (2) angiogenesis: angiogenesis is crucial for solid tumor growth and invasion (6). Inflammatory cytokines, such as TNF-α and IL-1, activate chemokine receptor-4/chemokine (C-X-C motif) ligand 12 (CXCR4/CXCL12) signaling, which upregulates vascular endothelial growth factor (VEGF) expression *via* the phosphatidylinositol 3-kinase/protein kinase B (PI3K/Akt) pathway. In addition to cytokines and chemokines, cyclooxygenases (COX)-2 and a portion of its metabolites are also engaged in vascular formation (31); (3) metastasis and invasion: inflammation also contributes to hypoxia, which further promotes angiogenesis, glycolysis, and invasion (31). Inflammatory cytokines secreted by immunosuppressive cells contribute to the progression of cancer. For example, MDSCs promote epithelial-to-mesenchymal transition (EMT) by secreting transforming growth factor β (TGF-β), epidermal growth factor (EGF), and hepatocyte growth factor (HGF) pathways (32) and shift M1 macrophages into TAMs (an M2 phenotype) (33). TAMs lose tumoricidal activity and contribute to immune suppression through the upregulation of inflammatory meditators [e.g., IL-10, TGF-β, and C–C motif chemokine ligand 22 (CCL22)], which promote T cell anergy and Treg recruitment (34). For further details, please refer to these current reviews that focus on inflammation in cancer development and progression (18, 29, 31).

## Anticancer Strategies Targeting the Inflammatory Microenvironment

### Antagonizing Inflammation

To date, several anti-inflammatory drugs have been used for prophylaxis and have shown efficacy in decreasing cancer morbidity (35), counteracting chemoresistance, suppressing tumor progression, and improving survival (10). Anti-inflammatory drugs are classified as non-steroidal anti-inflammatory drugs (NSAIDs) (e.g., aspirin), steroidal anti-inflammatory drugs (e.g., dexamethasone), or statins. In addition to its well-documented effects in CRC prevention (11), aspirin also reduces the incidence of several types of solid tumors, including melanoma (36), prostate cancer (37), and breast cancer (38). Mechanistically, aspirin inhibits the production of PGE_2_, a COX-metabolite derived from arachidonic acid (AA), which facilitates tumor growth through the enhancement of immune evasion (39). Aspirin was also adopted as a novel adjuvant to reverse chemoradiotherapy resistance (10, 40).

Steroids, such as dexamethasone and prednisolone, are widely used as monotherapies or combined with other therapeutic agents in various types of cancer. For instance, dexamethasone improves myeloma sensitivity to Venetoclax (a specific inhibitor of B-cell lymphoma-2) (41). In colon cancer, dexamethasone suppresses TGF-β1-induced migration *via* inhibition of AKT and extracellular signal-regulated kinase (ERK) phosphorylation (42). Dexamethasone is also used for the treatment of castration-refractory prostate cancer (43). The efficacy of statins has also been reported in a variety of cancers, such as HCC, CRC, and acute myelocytic leukemia (44).

Despite the multiple benefits of NSAIDs and steroids in cancer treatment, they have various adverse side effects, including gastrointestinal bleeding, liver and kidney dysfunction, Cushing’s syndrome, and osteoporosis (45–48). Some severe side effects of statins, such as necrotizing myopathy, increased risk of type 2 diabetes, and acute memory impairment, have also been reported (49–51). These negative side effects have restricted the full application of anti-inflammatory drugs to cancer therapy.

### Enhancing Antitumor Immunity

The development of cancer immunotherapy was a major milestone in current cancer treatments and ranked first on the list of the top 10 breakthroughs of 2013 in the journal *Science*. Recent developments in cancer immunotherapy include vaccines, cytokines, checkpoint-blocking antibodies, and immune cell adoptive transfer therapies. Typical cancer vaccines include cancer antigen vaccines, DC vaccines, and nucleic acid vaccines, among others. The melanoma-associated antigen 3 vaccine Stimuvax (targeting Mucin 1) has entered phase III clinical trials (52). Viral vector-infected or peptide-based DCs have been widely used to treat prostate cancer, glioma, melanoma, and CRC (52). Other adoptive cell transfer therapies, including cytokine-induced killer cells (53), tumor-infiltrating lymphocytes (54), gamma delta T cells (γδ T cells) (55), and NKT cells (56), are also being used to enhance clinical antitumor immunity.

Cytokines, such as IL-2, IL-18, IL-21, and granulocyte colony-stimulating factor (G-CSF), are also common adjuvants for cancer therapy. However, combination therapies have shown better curative effects. A recent phase III clinical trial in advanced melanoma demonstrated a significantly improved prognosis by combining a high-dose of IL-2 with a peptide vaccine (gp100) (57). In patients with non-Hodgkin’s lymphoma, combined treatment with recombinant human (rh)IL-18 and rituximab appeared to increase the overall objective response rate by 26.3% (58). A multicenter phase II study of patients with metastatic melanoma showed that IL-21 has antitumor activity (59). The combination of G-CSF and paclitaxel/carboplatin was validated for the treatment of patients with recurrent platinum-resistant ovarian carcinoma or recurrent or advanced endometrial or cervical carcinoma (60).

TNFerade is a genetically engineered adenovector with a radiation-inducible promoter that specifically delivers the human TNF-α gene to cancer cells. Phase I trials in patients with various tumor types (e.g., liver, breast, CRC, melanoma, sarcomas) confirmed that the combination of TNFerade and radiation was more effective than TNFerade or radiation alone (61). The phase I trial of TNFerade plus chemoradiotherapy also improved survival in patients with advanced resectable esophageal cancer (62).

Immune-checkpoint blockade is a revolutionary approach to cancer immunotherapy. Overexpression of programed death-ligand 1 (PD-L1) in tumor cells is correlated with poor prognosis, and immunotherapies with anti-PD-1/PD-L1 and anti-CTLA-4 antibodies have shown promising results in a variety of cancers (63, 64). Thus far, four antibodies have been licensed: (1) ipilimumab, an antibody against CTLA-4, has been licensed for unresectable or metastatic melanoma; (2) two mAbs against PD-1, pembrolizumab and nivolumab, have been approved for unresectable metastatic melanoma and advanced metastatic non-small-cell lung carcinoma. Nivolumab has also been approved for advanced (metastatic) renal cell carcinoma; (3) atezolizumab, a PD-L1-blocking mAb, has been used in metastatic or advanced urothelial carcinoma with platinum chemotherapy resistance (63). However, only a few cancers, such as lymphoma and melanoma, are sensitive to these antibodies because of the heterogeneity of cancers.

Recently, CAR-T immunotherapy, an emerging immunotherapeutic strategy, has achieved unprecedented success in cancer treatment. In CAR-T immunotherapy, T cells are modified to express specific receptors for the various types of cancer. Therefore, these T cells gain the ability to recognize and eliminate cancer cells after reinfusion into patients. A recent study showed that CAR-T cell therapy could mediate valid anti-leukemic activity in patients with acute lymphoblastic leukemia with chemotherapy-resistant B precursors and also exhibited feasibility and invertible toxicity (65). CAR-T therapy is also designed to treat chronic lymphocytic leukemia or B cell lymphomas, breast carcinoma, and glioblastoma (66, 67). However, CAR-T therapy is not widely used because it can induce the life-threatening cytokine release syndrome (CRS) (68) and has low efficacy against solid tumors.

Owing to the limitations of the abovementioned anti-inflammatory drugs and antitumor immunotherapies, it is urgent and essential to develop a novel, safe potent approach to conquer inflammation, and synergize the effects of immunotherapy in the treatment of cancer.

## The Potential Role of Inflammation Resolution in Remodeling the TME

### Failure to Resolve Inflammation Can Result in Cancer

Whether inflammation is a friend or a foe of cancer has always been controversial (5). As mentioned earlier, a failure of resolution promotes tumorigenesis, progression, and metastasis. TME is a complex environment including tumor cells, immune cells, fibroblasts, blood vessels, and the extracellular matrix (69, 70). It is widely accepted that TME reprograms immune cells into pro-tumor phenotypes with distinct metabolic and biological functions, which are required for the establishment and maintenance of tumors (8).

For instance, MDSCs are immature and immunosuppressive cells that also promote EMT *via* the TGF-β, EGF, and HGF pathways (32). The immunosuppressive properties of MDSCs are mediated by the following mechanisms: (1) l-arginine deprivation *via* upregulation of arginases; (2) ROS and reactive nitrogen species (RNS) generation; (3) restricting lymphocyte trafficking and viability; (4) promoting activation and expansion of Tregs; (5) shifting M1 macrophages to TAMs by producing IL-10; (6) inhibiting DC maturation and antigen presentation (33); (7) secreting MMPs, which facilitate tumor cell invasion *in vitro* and *in vivo* (71); and (8) regulating miRNAs in cancer cells, leading to enhanced stemness and metastasis potential (72).

Macrophages in the TME lose tumoricidal activity and contribute to immunosuppression through the upregulation of IL-10, TGF-β, and CCL22, which promote T cell anergy and Treg recruitment (34). During tumor initiation, TAMs exhibit activated glycolysis and inhibited oxidative phosphorylation (OXPHOSP). RNS, ROS, IL-β, and TNF-α are generated to drive genetic instability and promote cancer-related inflammation. Intriguingly, at the later stages of tumor progression, the energy metabolism of TAMs is skewed toward OXPHOSP by adenosine 5′-monophosphate (AMP)-activated protein kinase (AMPK) activation, lactate accumulation, IL-4 (from Th2 cells), and pyruvate kinase isozyme M2 activation (73). These metabolic changes drive the immunosuppressive phenotype in TAMs, which allows tumors to evade detection by the immune system.

In addition, nutrient exhaustion activates AMPK in TADCs, which promotes OXPHOSP, suppresses glycolysis and contributes to the immunosuppressive phenotype of DCs (74). TADCs elevate the numbers of Tregs and MDSCs in breast cancer, which in turn enhance bone metastasis by lowering the levels of CD8^+^ T cells (75). Recruitment of Tregs is also responsible for CD8^+^ T cell apoptosis and bone metastasis in breast cancer. Moreover, immune cell-derived TNF increases the infiltration of Tregs and MDSCs, which have been shown to enhance lung metastasis in a melanoma model (76). Recently, in experimental animal models of breast cancer, neutrophils have been identified as the main element and driver of metastatic formation within the premetastatic lung microenvironment, and neutrophil-derived LTs selectively expand the subpool of cancer cells with high tumorigenic potential in distant tissues (77). T cells carry out the bulk of immune surveillance; however, effector T cell activity is suppressed in the TME. The TME induces the loss of mitochondrial biogenesis in T cells, which drives metabolic insufficiency and dysfunction in tumor-infiltrating T cells (78). Moreover, the unresolved chronic inflammation aggregates low hydrogen ion concentration (pH) and hypoxia, accelerates extracellular acidosis, and further reprograms the metabolism of immune cells in the TME, which synergistically abrogates the efficacy of anticancer immunity (79). This evidence indicates a tight interaction between immune cells, and the TME that reprograms the plasticity of immune cells, suggesting that the failure of inflammation to resolve (chronic inflammation) can result in cancer.

### Inflammation Resolution and SPM

**Figure 3:**
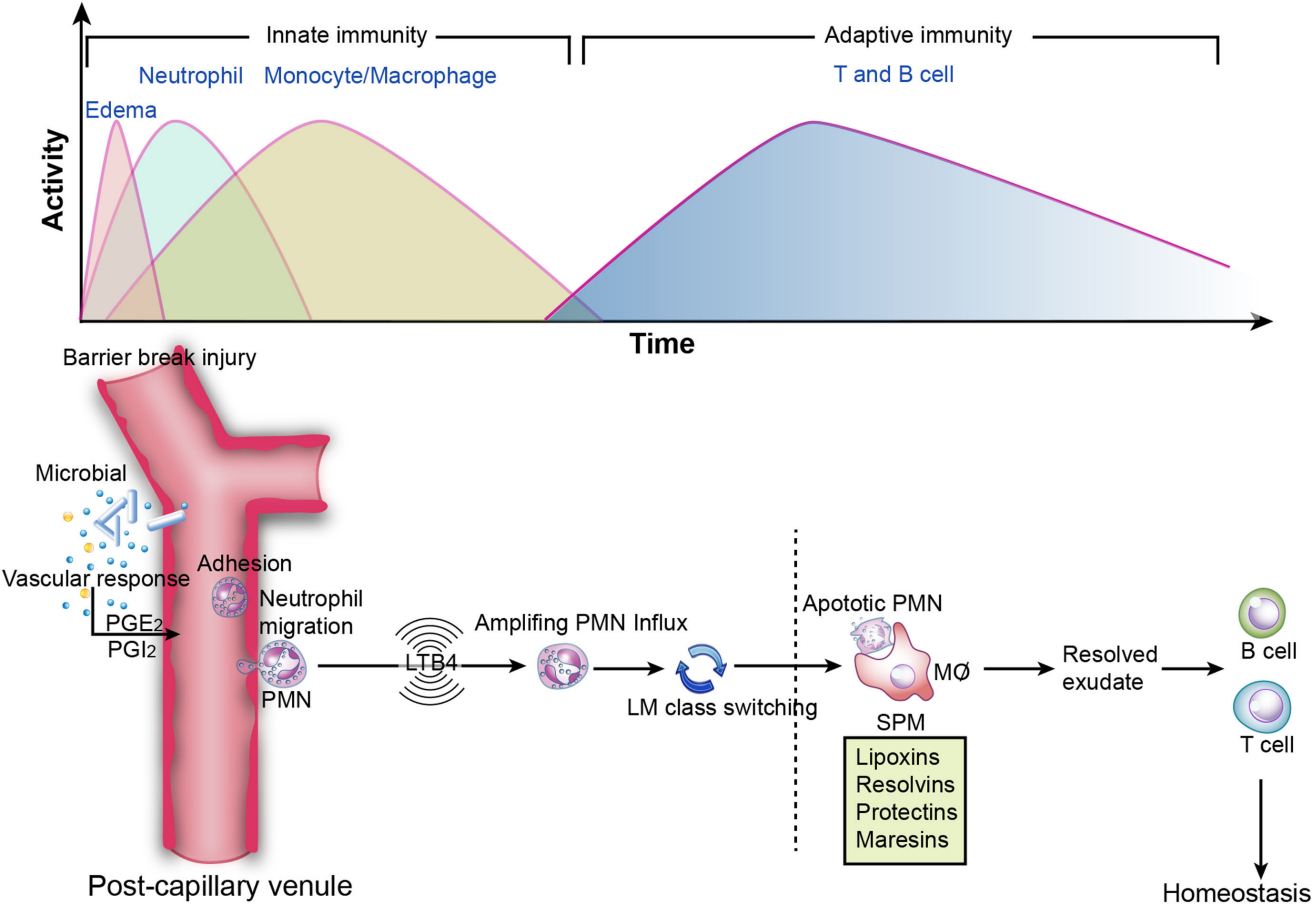
**Inflammation resolution**. Top image: innate and adaptive immunity in inflammation. During the initiation of inflammation, tissue edema is followed by polymorphonuclear neutrophil (PMN) influx and then a return to baseline, accompanied by the recruitment of monocytes and macrophages for resolution. Sequentially, effector T and B cells transform to memory T and B cells, which is essential for the secondary immune response. However, if resolution is not achieved, then the outcome is sustained inflammation (chronic inflammation). Bottom image: specialized pro-resolving mediators in the acute inflammatory response. PGE_2_ leads to vasodilation, and LTB_4_ stimulates PMN influx to the inflammatory loci. Subsequently, lipid mediator (LM) class switching converts pro-inflammatory signals to pro-resolving signals and triggers resolution. Lipoxins and resolvins restrict excessive PMN influx to the injury site, enhance efferocytosis, and stimulate pro-resolving signals and adaptive immunity.

Conventionally, inflammation is divided into two stages: initiation and resolution. The transition from resolution to homeostasis is an active process in acute inflammation orchestrated by SPM that possess versatile anti-inflammatory and pro-resolving properties (Figure 3) (1).

**Figure 4:**
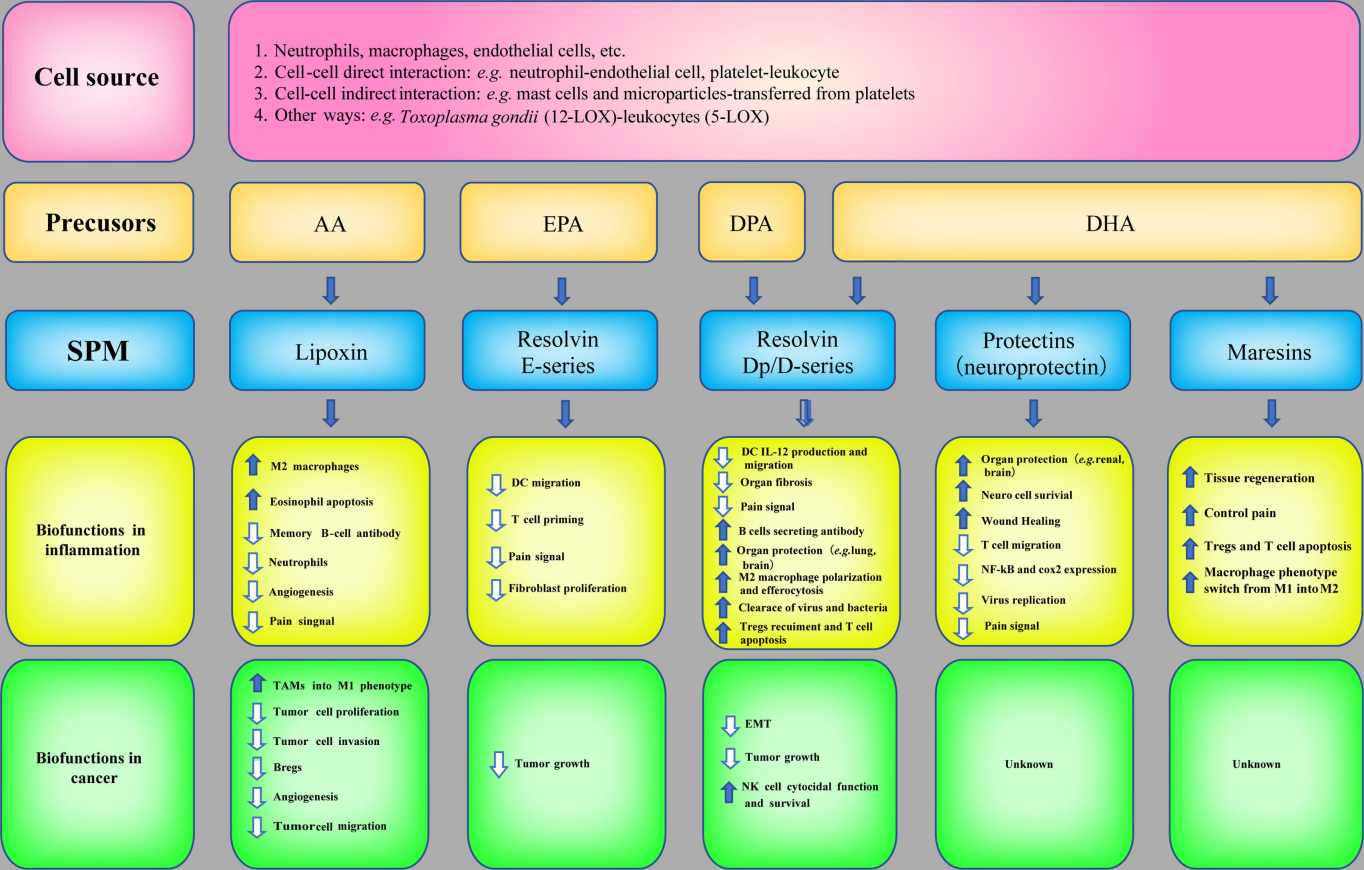
**Specialized pro-resolving mediators (SPM) in inflammation and cancer**. The biosynthetic pathways of SPM and their biofunctions in inflammation and anticancer immunity.

SPM are biosynthesized temporally from ω-3 and ω-6 poly unsaturated fatty acids (PUFAs), such as AA, eicosapentaenoic acid (EPA), docosapentaenoic acid (DPA), and docosahexaenoic acid (DHA), *via* the catabolism of lipoxygenases (LOX, e.g., 5-LOX and 12-LOX) and COX. LXs derived from AA were discovered by Serhan et al. (80). Later, LX epimers and aspirin-triggered LX (ATL) were identified (81). Omega-3 PUFAs, which are abundant in fish oils, can alter the expression of inflammatory genes and decrease the production of cytokines and the expression of adhesion molecules (82). The three major types of Rvs are series-D, Dp, and E-series. Specifically, RvD and RvDp are derived from DHA and DPA, respectively, RvE is generated from EPA and protectins and MaRs are derived from DHA. The protective actions of SPM have been demonstrated in acute inflammation [e.g., sepsis (83), lung injury (84), and ischemia reperfusion injury (85)] and chronic inflammation [e.g., asthma (86) and AD (87)]. The synthesis of SPM and their biofunctions in inflammation are summarized in Figure 4.

At the cellular level, the resolution of inflammation is characterized by the cessation of neutrophil infiltration and efferocytosis (macrophage clearance of apoptotic neutrophils). SPM restrict excessive PMN influx to the injury site and induce M1 macrophages to switch to the M2 phenotype, which confers improved phagocytosis abilities. Recent evidence has added a third phase of inflammation, termed post-resolution. During post-resolution, proliferation of memory T and B cells is increased. In this scenario, timely resolution of acute inflammation activates the priming and proliferation of T and B cells in the lymphatic tissues (88). Recent studies have reported that SPM regulate adaptive immunity *in vitro*. RvD1, RvD2, and MaR1 reduce the production of inflammatory cytokines (e.g., TNF-α and IFN-γ) in Th1 and Th17 cells while increasing the number of Tregs (89); LXA_4_ decreases memory but not naive B cell antibody production *via* an formyl peptide receptor 2 (FPR2/ALX)-dependent mechanism (90). These findings suggest that inflammation resolution links innate and adaptive immunity and that SPM play a role in both innate and acquired immunity.

### Anticancer Actions of SPM

Owing to the potent bioactivities of SPM in inflammation resolution and the correlation between inflammation and cancer, the roles of SPM in cancer have also attracted attention and investigation (Figure 4). The mechanisms are as follows:

(1) Directly targeting tumor cells: LXA_4_ shares structural similarities with estrogen 17-estradiol (E2) and possesses antiestrogenic ability *via* regulating estrogen receptors, indicating the therapeutic potential of LX in estrogen-associated diseases, such as endometrial cancer (91). LXA_4_ can significantly inhibit the proliferation and migration of lipopolysaccharide-stimulated HeLa cells *via* the nuclear factor-κB pathway. These effects can be abrogated by inhibiting its receptor, FPR2/ALX (92). In lung cancer, both RvD1 and RvD2 suppress TGF-β1-induced EMT by reducing the expression of zinc finger E-box binding homeobox 1 to prevent tumor metastasis (93). RvD1 induces high caspase-3 activity in pancreatic ductal adenocarcinoma cells (PDAC) *in vitro* (94).
(2) Targeting the TME: LXA_4_ and its analog dramatically inhibit the proliferation, invasion, and angiogenesis of hepatocarcinoma *via* remodeling the TME (13, 95); LXA_4_ is decreased in papilloma, and administration of LXA_4_ accelerates papilloma regression in mice (14); ATL treatment reduces the proliferation of lymphangioleiomyoma cells by inhibiting COX-2 (96). In human Kaposi’s sarcoma cells, LXA_4_ and ATL decrease phosphorylation of the VEGF receptor, ephrin family receptor tyrosine kinases, and pro-inflammatory mediators, including PGE_2_, LT B4, IL-6, and IL-8, to exert dramatic antiangiogenic actions (97). In murine xenograft tumor models injected with hepatocarcinoma, melanoma or colorectal carcinoma cells, LXA_4_ is able to suppress tumor growth by targeting IL-10-producing regulatory B cells (Bregs) *via* dephosphorylation of signal transducer and activator of transcription 3 and ERK. Since Bregs can cause CD8^+^ T cell dysfunction in the TME (12), these results suggest that LXs may reverse the CD8^+^ T cell response and improve antitumor immunity. Moreover, LX analogs inhibit VEGF-induced endothelial permeability by stabilizing the VE-cadherin/β-catenin-dependent adherens junctions to protect patients from tumor extravasation across endothelial barriers (15). An interesting recent finding revealed that LXs selectively switch M2 TAMs to an M1 phenotype, which triggers tumor cell apoptosis and blunts tumor progression (98). RvD1 protects NK cells against deactivation and increases NK cell cytocidal function in PDAC (94).
(3) Targeting precancerous lesions: LXA_4_ analogs block intestinal pro-inflammatory gene expression and inhibit the severity of colitis in a mouse model (99). An LXA_4_ isomer (10S, 17S-DiHDoHE) exerts an inhibitory effect on neutrophil infiltration and reduces pro-inflammatory cytokines, including TNF-α, IL-1β, and IL-6, thereby inhibiting the severity of colitis in mice (100). RvE1 increases survival and promotes resolution in a murine model of colitis (101). MaR1-induced attenuation of murine colitis was also recently observed in both dextran sulfate sodium and trinitro-benzene-sulfonic acid models (102). Furthermore, extensive clinical data have addressed the therapeutic role of omega-3 in various cancer types (e.g., breast cancer, CRC, leukemia, gastric cancer, pancreatic cancer, esophageal cancer, prostate cancer, lung cancer, head and neck cancer) and cancer cachexia (103). These trials suggest that SPM are the main mechanisms driving the antineoplastic effects of omega-3.

Together, the bioactions and mechanisms of SPM play important roles in attenuating tumor-promoting inflammation, which represents a synergistic principle that incorporates anti-inflammatory properties and enhances antitumor immunity. This new series of lipid mediators has created a potential new direction for cancer research.

## Conclusion and Prospects

Tumor growth is closely connected to inflammation, and the crosstalk between the two processes is context dependent. Inflammatory cell plasticity in the TME can promote or inhibit cancer. Cancer immunology and immunotherapy targeting the inflammatory microenvironment is an exciting field because it is at the brink of mainstream clinical practice and shows promising benefits. However, there are several substantial issues that need to be resolved. Therapy-induced inflammation often endows residual cancer cells with resistance to subsequent courses of treatment (e.g., chemotherapy resistance and radiotherapy resistance) (18). Moreover, the efficacy of immunotherapy depends on cancer types or populations. For example, CAR-T therapy is more effective in hematological neoplasms (65) than in solid tumors and may even lead to the development of life-threatening CRS (104). Although blockade of PD-1 elicits significant clinical benefits in patients with melanoma, some patients are innately resistant to anti-PD-1 therapy because of individual genomic and transcriptomic features (105). Aspirin restores the susceptibility of pancreatic cancer to gemcitabine (10) and reduces the risk of mortality in patients treated with radical prostatectomy or radiation for prostate cancer (106). Aspirin also acts synergistically with anti-PD-1 in tumor models (39). This evidence indicates that anti-inflammatory drugs may serve as useful adjuvants to conventional and immune-based therapies.

Presently, both cancer researchers and doctors have an important social responsibility to combat the increased incidence of tumors. The rapid development of oncology basic research, clinical diagnosis, and treatment provides more opportunities to overcome cancer yet also supplies more rigorous challenges. Targeting the TME is the current research focus; however, our ancestors have already provided us with some philosophical hints as to where we should focus our efforts.

In the primitive society of China, the Great Flood occurred and led to great misery in the people for many times. A superman by the name of Gun, who commiserated with his suffering people, tried to control the flood by blocking and damming. However, he failed. After his death, his son, Yu, carried on his father’s unfulfilled task, fighting against the Great Flood. For thirteen arduous years, he devoted himself conscientiously to his work. Drawing a lesson from his father’s failure, he used the methods of channeling and dredging and finally succeeded in subduing the Great Flood. In honor of his work, Emperor Shun asked him to take over the throne. Yu the Great is the personification of wisdom, perseverance and selfless devotion and, as such, he is a popular figure in artistic creations.

Controlling the cancer inflammatory microenvironment may be similar to controlling the Great Flood: the best strategy is not blocking but dredging. Thus, promoting endogenous pro-resolution factors may be a more safe and potent method for controlling the TME. However, the current evidence for the use of endogenous SPM in animal models with cancer is sparse, and the use of SPM in patients with cancer has not yet been investigated. Based on the versatile pro-resolving properties of SPM and the key roles of inflammation resolution in innate and adaptive immunity, we speculate that SPM may pave the way for the development of novel monotherapies or combination therapies that may provide a breakthrough in anticancer interventions.

## Author Contributions

YL conceived this topic and organized the manuscript. QZ and YL wrote the paper and drew the figures. BZ participated in the discussion and revision.

## Conflict of Interest Statement

The authors declare that the research was conducted in the absence of any commercial or financial relationships that could be construed as a potential conflict of interest.
